# Mapping policy gaps and governance challenges in Kenya’s hand hygiene environment: a health policy triangle analysis

**DOI:** 10.1093/heapol/czag092

**Published:** 2026-07-15

**Authors:** Naomi Njeri, Joash N Moitui, Blessing Mberu, Sheillah N Simiyu

**Affiliations:** Population Dynamics and Urbanization, African Population and Health Research Center, Manga Close, off Kirawa Road, 00100 Nairobi, Kenya; Population Dynamics and Urbanization, African Population and Health Research Center, Manga Close, off Kirawa Road, 00100 Nairobi, Kenya; Population Dynamics and Urbanization, African Population and Health Research Center, Manga Close, off Kirawa Road, 00100 Nairobi, Kenya; Population Dynamics and Urbanization, African Population and Health Research Center, Manga Close, off Kirawa Road, 00100 Nairobi, Kenya

**Keywords:** hand hygiene, Kenya, policy, health policy triangle, health, guidelines, strategy

## Abstract

Hand hygiene is an effective public health measure for preventing infectious diseases. Sustained hand hygiene practice requires an enabling environment that constitutes access to hand hygiene products and services, clear and effective policy provisions, financing, monitoring, and coordination mechanisms. This paper analyses Kenya's national hand hygiene policy landscape and identifies the underlying governance barriers and systemic opportunities for strengthening hand hygiene policy and practice in Kenya. Guided by the Health Policy Triangle (HPT) framework, this study systematically examined the context, content, process, and actors shaping Kenya's hand hygiene policy landscape. Primary data was collected through Key Informant Interviews (KIIs) with 11 strategic stakeholders involved in policy development, implementation, regulation, and programme delivery. Secondary data was through a comprehensive review of 20 policy instruments, including national policies, strategies, protocols, roadmaps, and binding legal documents. In Kenya, policies are formulated at the national level and devolved to the County level for implementation, with support from state and non-state actors. The analysis showed that Kenya lacks a standalone, hand hygiene policy document. However, hand hygiene provisions are present in policy documents which cover hand hygiene in several settings such as rural areas, schools, and health facilities. This reflects fragmented policy presence rather than complete absence. Barriers hindering effective implementation include weak inter-sectoral coordinating mechanisms, inadequate financing, and a lack of data. These gaps create governance and operational fragmentation, undermining the sustainability of hand hygiene interventions. To transition to an enabling environment, Kenya should establish explicit national hand hygiene standards with clear definitions and minimum requirements, secure dedicated, and sustainable financing mechanisms, ensure national-County coordination, and integrate standardized hand hygiene indicators into the national and sub-national health information systems. These reforms will support more coherent governance, evidence-informed planning, and accountability for hand hygiene implementation.

Key messages

- Globally, hand hygiene is recognized as a cornerstone of public health, yet it is often bundled with broader water and sanitation initiatives; which can make hand hygiene administratively visible but weakly prioritized in financing, implementation, and monitoring.
- This study provides a comprehensive analysis of hand hygiene policy landscape in Kenya using the Health Policy Triangle framework. Analysis reveals that decentralized governance structures create opportunities for lower-level implementation, but the lack of a dedicated hand hygiene policy in Kenya with responsibility shared amongst stakeholders provides gaps especially in accountability.
- These findings highlight the importance of strengthening accountability frameworks at sub-national levels, and that sustainability of hand hygiene relies on having a dedicated budget code in place, with policymakers integrating hand hygiene indicators into national and sub-national health information systems.

## Introduction

According to the World Health Organization (WHO) and the United Nations Children’s Fund (UNICEF), hygiene encompasses a diverse range of practices that include hand hygiene, food hygiene, menstrual hygiene management, oral hygiene, and environmental cleaning; and due to this broad scope, there is currently no universally accepted definition of hygiene (UNICEF and WHO 2021). Hand hygiene refers to behaviours that clean hands and include handwashing with soap and water, as well as the use of alcohol-based hand rubs (UNICEF and WHO 2021). In this manuscript, hygiene is an umbrella term encompassing diverse public health practices that maintain cleanliness and prevent disease; hand hygiene is a distinct behaviour referring to actions of cleaning hands; and handwashing or handwashing with soap is a specific hand hygiene method of using soap and running water.

Hand hygiene is effective in the prevention and control of infectious diseases such as diarrhoea and respiratory infections (Alzyood et al. 2020, Wolf et al. 2022, Ross et al. 2023). Hand hygiene should be practiced as much as possible and in various settings including domestic spaces, health care settings, workplaces, schools, public spaces or spaces of mass gathering, and in dislocated populations (Cronk et al. 2015, Cronk and Bartram 2018, World Health Organization 2020, Berendes et al. 2022, de Kraker et al. 2022). As such, the Joint Monitoring Programme of the WHO and UNICEF monitors progress in achieving hand hygiene goals in domestic spaces, health care facilities, and schools (WHO and UNICEF 2021, 2024, UNICEF and WHO 2024). The key indicator for tracking hand hygiene is access to handwashing facilities with soap and water (WHO and UNICEF 2021).

The UNICEF and WHO note that effective hand hygiene requires an enabling environment, equitable access to hand hygiene products and services, and evidence-based behaviour change interventions, all of which depend on effective political leadership (UNICEF and WHO 2021). In line with the Sustainable Development Goals (SDGs), nations aim to have most of the populations in their countries with access to basic hygiene services. Some suggested interventions to achieve hand hygiene for all include establishing hand hygiene definitions, capacity development of key staff, adequate financing, encouraging innovation, and establishing hand hygiene policies with established coordinating mechanisms (UNICEF and WHO 2021). It is also recommended that sustainable hand hygiene systems require national systems and plans that are integrated within existing programmes and are driven by governments and the health sector (Esteves Mills et al. 2023; WHO and UNICEF 2025b).

At a national level, policies guide programme implementation to achieve national goals, and their absence can result in uncoordinated implementation of programmes which hinders sustainable development (Auriacombe and Van der Walt 2021). Specific to hand hygiene, the lack of policies has been identified as one of the barriers for effective hand hygiene among health care providers (Afework and Tamene 2025), and where policies and guidelines exist, there has been notable compliance and strengthening of hygiene promotion (Choe et al. 2020). A review of national hygiene policies and plans conducted across several countries revealed that countries generally have national hygiene policies but are constrained by financial and human resources, insufficient funding for hygiene, and limited data (WHO 2020). Moreover, the COVID-19 pandemic may have led to the development of several policies and guidelines to promote hand hygiene.

Implementation of policies in Africa is often hindered by factors such as fragmentation across actors, unclear agenda, and inadequate evidence (Ansah et al. 2024). Such challenges may hinder the effectiveness of policies in achieving national goals and targets. Several hygiene interventions have been implemented in Kenya in different settings and there has been progress in access to basic hygiene services, with the most recent statistics indicating that 58% of the population have access to basic hygiene services (WHO and UNICEF 2025a). However, there is insufficient evidence on the hand hygiene policy landscape, how policy responsibilities are distributed across national and County institutions, and why gaps in coordination, financing, and monitoring persist despite the presence of multiple Water, Sanitation, and Hygiene (WASH)-related policy instruments. This manuscript therefore, moves beyond a descriptive mapping and provides a comprehensive diagnosis of the hand hygiene policy landscape in Kenya. The manuscript systematically evaluates the hand hygiene policy landscape, explains how institutional arrangements, actor relationships, and policy processes shape hand hygiene governance and practice; and reveals the underlying barriers and systemic opportunities for effective hand hygiene governance and practice in Kenya.

## Materials and methods

### Theoretical framework and approach

This analysis adopted the Health Policy Triangle (HPT) framework, an approach used to analyse policies by examining the context, content, processes, and actors involved in particular policies (Walt and Gilson 1994). Context refers to systemic factors such as social, economic, political, cultural, and other environmental conditions, while content refers to policy objectives, operational policies, legislation, regulations, and guidelines. Actors are influential individuals, groups, and organizations involved in the policy process, and process refers to the way in which policies are initiated, formulated, negotiated, communicated, implemented, and evaluated (Walt and Gilson 1994). The framework can be applied retrospectively or prospectively and it has been used to understand policy experiences across different contexts (O’Brien et al. 2020). The HPT was used analytically rather than only descriptively. We examined how the devolved governance context shaped implementation processes, how national and County actors influenced policy content and priority-setting, and how the absence or presence of specific policy provisions affected financing, coordination, and monitoring. This approach allowed the analysis to move beyond identifying the existence or absence of hand hygiene provisions, towards explaining how governance arrangements, institutional mandates, and implementation processes interact to produce policy gaps. Contents of the policy frameworks in relation to hand hygiene were reviewed by adopting a conceptual framework for hand hygiene developed by MacLeod et al. (2023). The framework reviews hand hygiene across four domains: Effective hand hygiene, minimum requirements, behaviour change, and government measures. We modified the framework and reviewed content across four areas: Effective hand hygiene (covering definitions, critical moments, and methods of hand hygiene), requirements for hand hygiene, settings (e.g. schools and public spaces), and strategies for effective hand hygiene. Using this approach with the HPT framework enabled triangulation and supported analysis of interactions between policy content, governance context, actors, and implementation processes, thus strengthening the explanatory power of the findings. The HPT was the main framework guiding the analysis, while the modified MacLeod *et al*. framework was nested within the content domain of the HPT framework to systematically analyse the technical hand hygiene provisions within the documents.

### Study design

The study adopted a concurrent qualitative study design that combined primary and secondary data sources. Primary data collection was through semi-structured Key Informant Interviews (KIIs) with relevant stakeholders, with the aim of understanding existing hand hygiene policies and their implementation, while secondary data collection entailed a systematic tracking and review of existing hand hygiene-related policy and legal instruments. This concurrent approach enabled continuous and iterative cross referencing between documents and stakeholder perspectives. Importantly, policy instruments cited by key informants were cross checked against the documents generated from our search, and served as a completeness check that allowed a comprehensive review of key regulatory frameworks governing hand hygiene in Kenya.

### Primary data collection: key informant interviews

Two-stage purposive and snowball sampling approaches were used to select respondents. An initial stakeholder list was compiled from membership of national WASH Technical Working Groups (TWGs) and engagement meetings during prior sector engagements. Notably, the study targeted national level policies, but the target population of policy experts in hand hygiene policies is small. From this directory, an initial list of five key informants was selected based on their roles, responsibilities, and/or expertise in hygiene policy, WASH governance, implementation, regulation, or service delivery. These stakeholders included representatives from the relevant ministries, policy experts, and implementers of hand hygiene interventions. A snowball approach was then adopted where the initial informants suggested other suitable stakeholders who were then included in our list and followed up for interviews. To reduce over-reliance on a single network, we sought variation across national ministries, County-level implementation structures, policy and legal experts, implementers of WASH interventions, and regulatory bodies. Selection of respondents continued until new interviews were no longer generating substantially new information on the policy instruments, institutional roles, coordination challenges, financing constraints or data gaps that were central to the study objectives.

Interviews were conducted in-person or virtually through online platforms. All interviews were conducted at a time convenient to the informants, with in person interviews being conducted at venues convenient to the stakeholders. The Interviews were conducted in English by at least one experienced interviewer and a note taker who followed an interview guide that had questions focusing on hygiene policies, projects and partnerships, monitoring and evaluation, challenges, and recommendations. The interviews were audio recorded in order to capture all information, and they lasted about 1 h. A total of 11 key informants who included legal and governance experts (2), representatives from relevant ministries at the national and County level (*n* = 4), County level staff who were directly responsible for implementation of activities (*n* = 2), implementers of WASH interventions (*n* = 2), and representatives from regulatory bodies (*n* = 1) were interviewed. County level staff were selected from Nairobi, Nakuru, and Kisumu Counties where ethical approval for the research was obtained.

### Secondary data collection: systematic document review

To identify relevant policy documents and guidelines, a systematic search of grey literature and official databases was conducted. The search was restricted to policy instruments published between 2010 when the Constitution of Kenya was promulgated and 2024. To ensure completeness, the search was conducted across multiple platforms, including: Official Government Portals of the Ministry of Health (MoH), Ministry of Water, Sanitation, and Irrigation (MoWSI), and the National Environment Management Authority (NEMA); Legal Databases such as the Kenya Law Reports (National Council for Law Reporting) for acts, bills, and regulations; and repositories and Search Engines including Google Scholar and Google Search Engine using defined search terms. These search terms and combinations included: ‘hand hygiene policies Kenya’, ‘handwashing Kenya’, ‘handwashing with soap Kenya’, ‘handwashing policies Kenya’, ‘water, sanitation hygiene policies Kenya’, ‘hand hygiene programmes in Kenya’, and ‘handwashing programmes in Kenya’. Acknowledging that ‘hand hygiene’ and ‘handwashing’ are often used interchangeably even within literature, these terms were intentionally included in the search terms to minimize the risk of omitting some documents thus ensuring completeness. The inclusion criteria were documents such as national policies, strategic plans, acts, and bills that were specific to Kenya, published from 2010, and which contained explicit or implicit provisions related to hand hygiene, handwashing, hygiene facilities, hygiene promotion, water, sanitation, and hygiene in schools or health facilities, rural sanitation, and hygiene, or relevant institutional mandates. Documents focusing on WASH were included only if they contained components of hygiene. Documents were excluded if they were not specific to hand hygiene, were project-only materials without policy or implementation guidance, were sub-national documents that were not linked to national frameworks, or documents published before the 2010 Constitution. Although the water and sanitation aspects of WASH were not the main focus of the review, water and sanitation documents were included if they contained provisions that shaped hand hygiene requirements, financing, infrastructure, coordination or monitoring.

Titles and executive summaries were first screened by two independent reviewers to verify their relevance. Once deemed relevant, these documents were shortlisted and the full-texts were reviewed. To ensure completeness, further analysis of the references in the reviewed documents were performed until no new policy documents emerged. Twenty policy instruments were reviewed, and these were classified as policies (*n* = 7), strategies (*n* = 3), guidelines (*n* = 1), strategic plans (*n* = 1), protocols (*n* = 1), roadmaps/policy frameworks (*n* = 2), and cross-sectoral development plans (*n* = 1). In addition, four binding legal instruments (Constitution, Acts) were reviewed.

### Data analysis

Our analytical approach was guided by the HPT framework by examining the four domains of actors, process, context, and content in each of the policy documents and the key informant interviews. To ensure transparency in our analysis, an inductive-deductive coding process was used. Initial open codes were generated from the documents and transcripts. Audio recordings from the qualitative interviews were transcribed to Microsoft Word for analysis. Two researchers read through the transcripts to identify key words and codes related to policy formulation, institutional roles, coordination, financing, implementation, monitoring, and policy gaps. The researchers then compared codes, discussed differences, and developed an agreed coding framework for the full analysis. Policy documents were read through to identify words or phrases related to hand hygiene, such as handwashing, handwashing with soap, hand hygiene, handwashing facilities, and hygiene facilities. This list formed the coding framework that was used for analysis. Codes were then grouped into sub-themes such as handwashing infrastructure or institutional roles. Further analysis entailed reading through the documents to identify sub-themes and their relationships. The sub-themes were deductively mapped onto the overarching HPT domains and findings from interviews and documents were triangulated by comparing what policy instruments specified with how key informants described implementation in practice. To understand content, analysis explored aspects such as information on hand hygiene definitions and practices (e.g. when and how hand hygiene should be practiced), information on infrastructure, products, and services necessary for effective hand hygiene, provisions for hand-hygiene in places where people learn, work, stay, or visit; and information on measures taken to ensure effective hand hygiene as per the modified MacLeod *et al.* framework. These were then mapped into sub-themes such as effective hand hygiene practices, basic requirements for hand hygiene, settings, and actionable strategies. This analytical approach is presented in Table 1. Results from the two data sources were incorporated in the presentation of results and discussion, ensuring a detailed assessment of Kenya’s hand hygiene policy landscape.

**Table 1:** Data analysis matrix along the four domains of context, actors, process and content.

HPT domain/theme	Definition	Coding/sub-themes derived from data	Examples
Context	Systemic factors such as social, economic, political, cultural, and environmental conditions shaping hand hygiene policies	Political devolution Socio-economic disparities Policy formulation process	Devolution of health functions to counties. Implementation of policies at Counties Inclusion of population groups in policy formulation
Actors	Individuals, groups, and organizations involved in policy process	Institutional roles (Ministries) State and non-state actors Development partners Community level actors	Roles of the different ministries in policy formulation and implementation
Process	How policies are initiated, formulated, negotiated, communicated, implemented, and evaluated	Policy formulation barriers Multi-sectoral coordination Monitoring and evaluation mechanisms	Process of policy development. Implementation at county level
Content (Analysed via modified MacLeod *et al*. framework)	Specific provisions and scope of the policy instruments.	Effective Hand Hygiene: Definitions, critical moments, and methods. Requirements: Infrastructure, products (soap/sanitizer), and services. Settings: Schools, public spaces, healthcare facilities, and workplaces. Strategies: Actionable behaviour change campaigns, funding allocations.	Specific clauses stating or defining hand hygiene, definition of proper handwashing with soap Clauses defining roles of institutions and stakeholders in hygiene provision

## Results

The reviewed documents are listed in Table 2, and are categorized into the legal and policy instruments. Policy instruments/frameworks are guidelines for national level decision-making and programme implementation while legal frameworks are national binding laws and regulations that provide an enabling environment and structure for the policy frameworks.

Policies direct individuals on how to behave in particular setups, and with regards to something…they identify a problem and make proposals on how to address the issue. There are no legal sanctions for non-adherence to policies. Laws are what you are supposed to do and failure has legal consequences. [Legal expert]

Most of these policy and legal instruments were mentioned by the key informants as documents that had components of hand hygiene.

**Table 2:** Legal and policy instruments relevant to hand hygiene in Kenya and their scope on hand hygiene requirements.

	Policy document	Document type	Objective	Settings covered	Defines hand hygiene	Implementation guidance	Financing provisions	Monitoring indicators
1	The Kenya Environmental Sanitation and Hygiene Policy 2016–30	Policy	Provide a framework to improve environmental sanitation and hygiene to promote health and well-being in Kenya.	Household, Community, Schools, health care facilities, institutions, public spaces	Hand hygiene is not defined. Hygiene is the umbrella term and key behaviour that includes handwashing with soap at critical times. Handwashing is a hygiene behaviour.	Specifies handwashing promotion strategies, role of counties, use of Behaviour Change Communication (BCC), and integration with WASH programming.	It calls for government, county, private sector, and development partner financing; it does not specify dedicated hand hygiene or handwashing budget lines.	It includes hand hygiene indicators such as % households with handwashing facilities with soap and water as part of broader WASH Monitoring and evaluation framework. These indicators capture availability, not behaviour.
2	Kenya Environmental Sanitation and Hygiene Strategy 2016–30	Strategy	Operationalises the environmental sanitation and hygiene policy by providing roles, strategies, and responsibilities for different stakeholders.	Household, Community, Schools, health care facilities, institutions, public spaces	Hand hygiene is not defined, but hygiene is noted as a key behaviour that includes handwashing with soap at critical times. Hand hygiene and handwashing with soap are used interchangeably.	Provides detailed implementation actions for handwashing promotion including Social and Behaviour Change Communication, supply-side interventions, community mobilization, and integration with ODF campaigns.	It identifies government and County budget allocations and donor funding mechanisms but lacks dedicated hand hygiene cost estimates.	It includes specific handwashing indicators (e.g. % households with functional handwashing station with soap) tied to strategy targets and timeframes.
3	The Kenya School Health Policy	Policy	Promote well-being of school-going children by ensuring students access WASH facilities, have adequate water supply, menstrual hygiene management, and hygiene education to promote good hygiene practices.	Pre-primary, primary and secondary schools	References handwashing and hygiene promotion but does not provide a definition of hand hygiene. Handwashing is listed as a hygiene practice.	Specifies roles of teachers, school health clubs, and county health teams in provision of handwashing facilities and hygiene education.	Financing responsibilities are shared between Ministry of Education, counties, and school development committees; no specific hand hygiene budget.	School health indicators include WASH infrastructure in schools; hand hygiene-specific indicators are not disaggregated. Specific indicators capture availability.
4	The Kenya Community Health Policy	Policy	Provides a framework for community health services delivery and outlines WASH roles for the community health personnel.	Household and communities	Handwashing is listed among CHV roles, and is implied within hygiene promotion activities. Hand hygiene is not formally defined.	CHPs are tasked with household hygiene promotion including handwashing messaging, but detailed hand hygiene protocols are not specified.	Community health fund and county health budgets are referenced activities; but hand hygiene is not costed separately.	Household WASH indicators are monitored at community unit level and handwashing with soap is one of hygiene behaviour indicators.
5	Policy Guidelines on Control and Management of Diarrheal Diseases in Children Below Five Years in Kenya	Guidelines	Provides a framework for prevention and control of diarrhoea morbidity and mortality of children under the age of 5 years in Kenya.	Households and communities	No definition is provided, handwashing with soap is identified as a key preventive measure; critical times of handwashing as per the WHO are referenced.	Guidance on handwashing as a preventive intervention is provided; including promotion and integration with treatment protocols.	Financing for hand hygiene is not discussed	Although surveillance indicators for diarrhoea morbidity/mortality are provided, hand hygiene is not a specific indicator.
6	Menstrual Hygiene Management Policy	Policy	Promotes healthy and dignified menstrual hygiene management for women and girls.	Schools, communities, health facilities, workplaces	Hygiene is the umbrella term; hand washing is referenced in the context of menstrual hygiene practices; hand hygiene is not defined.	Hand washing is mentioned as part of MHM practice guidance, but handwashing protocols are not elaborated.	Financing is mentioned through County governments, MoH, MoE, and development partners but it is not disaggregated for hand hygiene components.	Menstrual hygiene management indicators include handwashing; but hand hygiene is not a standalone indicator.
7	Menstrual Hygiene Management Strategy	Strategy	Provides implementation strategies for the policies stipulated in Menstrual Hygiene Management Policy	Schools, communities, health facilities, workplaces	Hygiene is used as an umbrella term. Handwashing is referenced within menstrual hygiene management practices. Hand hygiene is not defined.	Handwashing is included in MHM practice guidance that focuses on SBCC, supply of WASH facilities, and capacity building.	The document references government and donor financing but hand hygiene is not costed.	The monitoring framework includes WASH-related MHM indicators; but hand hygiene is not disaggregated as a separate indicator. Handwashing is captured among MHM indicators.
8	Costed Kenya Rural Sanitation and Hygiene Roadmap	Roadmap (Policy Framework)	Provides a roadmap to achieve access to rural sanitation and hand hygiene	Rural households, communities, schools	Hygiene is defined, hand hygiene is not explicitly defined, but is referenced as a distinct behaviour. Handwashing is defined as part of hand hygiene as a key behaviour.	Implementation strategies include Community Led Total Sanitation (CLTS), social norms approaches, supply chain strengthening for soap and handwashing facilities, and community-led monitoring. Critical handwashing times are operationalized.	Provides costed estimates for achieving hand hygiene targets; identifies financing gaps, government, county, development partner, and private sector funding requirements. Proposes distinct financing of hand hygiene	Specified indicators include % of rural households with basic handwashing facility with soap and water. Distinguishes access indicators from behaviour indicators.
9	The National ODF Kenya 2020 Campaign Framework	Framework	Provides Open Defecation Free (ODF) campaign frameworks for improved rural sanitation and hygiene.	Rural communities, households, schools	Hand hygiene is not explicitly defined. Handwashing with soap is used as part of hygiene as a key behaviour.	Handwashing promotion included as part of ODF criteria	County and national government financing is referenced; hand hygiene financing not separately costed.	ODF verification includes presence of handwashing facility with soap/ash near latrine as part of criteria.
10	Kenya Infection Prevention & Control Strategic Plan (2021–25)	Strategic Plan	Strengthen infection prevention and control in health care facilities to reduce healthcare-associated infections.	Health facilities	Hand hygiene is noted as a key element of infection prevention, and is distinguished from broad hygiene, although in clinical settings. It recognizes multiple hand hygiene methods including use of alcohol-based hand rub.	Guidance is provided on IPC strategy implementation including moments of hand hygiene, hand hygiene promotion campaigns, placement of hygiene facilities, formation of committees, and development of standard operating procedures.	Resource mobilization strategies include MoH budget, counties, development partners. Alcohol-based hand rubs and hand hygiene supplies mentioned as priorities. Hand hygiene budget is not explicitly costed.	Hand hygiene compliance rates are core IPC indicators; periodic audits and WHO Hand Hygiene Self-Assessment Framework referenced. Indicators are behaviour based.
11	National Sanitation Management Policy	Policy	Provides a comprehensive framework for universal, safely managed sanitation services by 2030.	Household, community, schools, public spaces, urban and rural areas	Hygiene is referenced as part of sanitation and behaviour change but is not explicitly defined. Handwashing behaviour is linked to sanitation. Hand hygiene is not defined.	Handwashing included in sanitation management activities; implementation is delegated to county governments.	Outlines national and county government financing roles; but hand hygiene is not costed.	Hygiene indicators including access to handwashing facilities are referenced but they are not disaggregated to hand hygiene behaviour compliance.
12	National Water and Sanitation Services Strategy (2020–25)	Strategy	It guides the national water and sanitation services sector to achieve sustainable access to water and sanitation services.	Urban and rural households, institutions, public spaces	Hygiene is noted as linked to water and sanitation services but is not defined. Hand hygiene is also not defined. Hygiene is treated as an outcome of access to water and sanitation.	Hygiene promotion including handwashing is mentioned as an intervention but guidance is absent.	Water and sanitation financing mechanisms are addressed but hand hygiene is not separately financed or costed. Hand hygiene is assumed as an outcome of water and sanitation services.	Hand hygiene behaviour not a primary indicator. Hygiene outcomes are implied through access to water and sanitation services.
13	The Kenya National Environment Policy	Policy	Provides a framework for managing the environment and natural resources in Kenya.	National	Hand hygiene is not addressed.	Has environmental health provisions related to waste management but lacks hand hygiene guidance.	Environmental financing is discussed but not related to hand hygiene.	Environmental indicators do not include hand hygiene.
14	Kenya Health Policy (2014–30)	Policy	This document provides an overall long-term health sector direction for achieving the highest attainable standard of health.	National-all health settings and levels of care	Hygiene is referenced as a preventive health measure but no definition is given. Handwashing is implied within hygiene promotion.	Implementation of hygiene activities is delegated to sector-specific strategies.	Financing principles include government, county, private, and development partner contributions, but hand hygiene is not separately costed.	Hand hygiene is not a specific indicator. Hygiene is assumed within broader health monitoring.
15	Rural Sanitation and Hygiene Protocol	Protocol	Provides guidance for the implementation and achievement of improved sanitation and hygiene in rural communities in Kenya.	Rural households, communities, schools	Hygiene and handwashing with soap are defined. Hygiene is the umbrella term and handwashing with soap is recognized as a specific behaviour.	Implementation for hygiene promotion including behaviour change approaches is provided, for example, SBCC, construction of tippy taps, and soap provision.	Implementation costs are discussed but hand hygiene financing is not costed; community contributions and government inputs are referenced.	Hygiene behaviour indicators including handwashing facility availability and use at household and community levels are included.
16	Kenya Vision 2030	Development Plan	A national development plan for the country that aims to transform Kenya into a middle-income country providing high quality of life to all citizens by 2030.	National	Hand hygiene is not specifically addressed.	Sector-specific implementation guidance for hand hygiene is not provided.	Although development financing frameworks are referenced, they are specific to health and water sectors.	No specific hand hygiene indicator.
	**Legal frameworks**							
17	The Constitution of Kenya	Constitution	Guarantees rights of citizens and the governance framework in Kenya including rights to sanitation, health, and clean water, establishes the devolved government framework.	National	Hand hygiene is not defined.	Not defined. Implementation through County governments.	The recognition of the right to health creates an obligation for government financing of services.	Not specified.
18	The Water act	Act (Law)	Makes provisions for the equitable and sustainable use and management of water resources and water supply.	National	Hand hygiene is not defined.	It provides a framework for water supply but does not mention hand hygiene	Establishes financing mechanisms for water services but hand hygiene is not directly addressed.	Hand hygiene is not included.
19	The Public Health Act, Cap 242	Act (Law)	Provides guidelines for prevention and control of communicable diseases and protection of the health and well-being of the population.	National	Hygiene requirements are referenced and implied but hand hygiene is not defined.	Empowers health officers to enforce hygiene standards including provisions on food handling, water quality, and sanitation.	Not specified.	Hand hygiene is not specifically monitored.
20	County Government Act 2012	Act (Law)	Defines the governance, management, and functions of County governments, including devolved services such as WASH.	County level	Not defined	Not defined, but mandates Counties to implement health and sanitation services.	Resources from national and County level generation mechanisms should enable counties to finance services including hand hygiene.	Hand hygiene indicators are not specified.

BCC-Behaviour Change Communication, CHP-Community Health Promoter, CLTS-Community Led Total Sanitation, IPC-Infection Prevention and Control, MHM-Menstrual Hygiene Management, MOE-Ministry of Education, MOH-Ministry of Health, ODF-Open Defecation Free, SBCC-Social and Behaviour Change Communication, WASH-Water Sanitation and Hygiene

To provide a comprehensive analysis of hand hygiene in Kenya, presentation of results is framed around the four domains of the HPT. The first three domains of context, process, and actors detail the structural, systemic, and institutional landscape of policy-making. The final domain of content is further categorized into the four dimensions of the modified MacLeod *et al*. framework. Consequently, the content domain is more detailed and occupies a large share of the findings.

### Context

Under the 2010 constitution, Kenya has two levels of government—National and County governments, operationalized under the Kenya County Government Act (GOK 2010, Republic of Kenya 2012). The Act provides a legal framework for the operationalization of the decentralized system of government and regulates the functions and responsibilities of the County governments. According to the constitution, the national government is responsible for national level functions including policy formulation, capacity building, technical assistance, and standard formulation; while County governments are responsible for service delivery, policy implementation, and infrastructural development. County governments also raise funds through local sources such as taxes and fees for services, while also receiving an equitable share of resources from the national government (GOK 2010). County governments further decentralize their functions and service delivery to other lower levels including city/town, Sub-County, and Ward for effective service delivery (Republic of Kenya 2012). Hygiene service provision, therefore, falls with County governments, while policy formulation rests with national government.

The role of County governments is for functions that are devolved, and water, sanitation, and hygiene are devolved functions. That means County governments are responsible for service provision and national governments are responsible for policy formulation. [Policy expert]

### Process

The policy development process in Kenya goes through several stages, including problem identification and agenda setting, formulation, approval, and implementation; which are then followed by monitoring and evaluation that may call for review of the policies (Republic of Kenya 2024). This process was reiterated from the interviews with stakeholders.

Agenda setting is the first step which is followed by policy formulation. After policy formulation, we have adoption of the policy, then implementation, and evaluation of the policy [Legal expert].

During problem identification, stakeholders specify the problem, its magnitude, causes, and those affected. Interviews further indicated that identification of the problem can be from various sources.

The need could be from the government, stakeholders, or from consumers. For example, if people are unable to access public toilets, that is a need. For government to provide these facilities, there must be a policy framework. [Policy expert]

Policy formulation entails drafting the policy document, and taking into consideration factors such as political, social and technical feasibility, equity, efficiency, and effectiveness. The draft is subjected to public participation where comments from the public are incorporated, and a validation workshop is held where the draft team explains how the comments have been incorporated. The policy is then formally accepted and implemented. Monitoring and evaluation of the policy entails data collection and assessment of the effectiveness of the policy-lessons that are documented and which can be used to review the policy.

The policy formulation process involves several sectors including development partners, civil society organizations, research and academic organizations, the public, the judiciary, and the national and County assemblies. The cabinet approves public policies (Republic of Kenya 2024). Often times, the drafting of a policy is led by a task force and a Technical Working Group that comprises experts in the subject matter who are drawn from relevant sectors.

A steering committee was set up, a consultant was hired, consultations were done across the country… the ideas were put together in a document, and validation was done. A document was written and experts looked at it, then finally became a draft document. You can't skip any one step. [Policy expert]

Discussions with Key informants also revealed the importance of being inclusive in policy formulation.

Ensure to include everyone … research organizations or learning institutions, women representatives, youth…, if the provisions [of the policy] cater for the needs of certain groups then their voices should be heard. [Policy Expert]

Once policies are formulated and approved, County governments contextualize and implement the policies in their respective Counties. Discussions with key informants also revealed that Counties can create guidelines at the County level, but they should align with national policies.

A County can only come up with policies that conform to the national government policy. For example, A County may have a [policy] document on sanitation which the national government may not have. In that case, when the national government comes up with a policy, the County policy should never contradict national policies. [Policy Expert]

### Actors and institutional frameworks

The policy documents highlight the role played by various actors in development and implementation of hygiene-related policies. In Kenya’s devolved governance system, national ministries are primarily responsible for policy formulation, standard-setting, and technical guidance. Since hygiene is associated with water, sanitation and health, hygiene-related provisions are covered in policy documents aligned to water and Sanitation. In 2019, the MOWSI was appointed to be responsible for sanitation. As such, the ministries of Water, Sanitation, and Irrigation, and the MoH have responsibilities related to hygiene in their policy documents. Policy documents developed after 2019 specify that the MOWSI is confined to urban areas while the MoH is responsible for sanitation and hygiene provision in rural areas and consequently leads policy development and implementation that covers hygiene in both rural and urban areas (Ministry of Health 2023). However, the Kenya Environmental Sanitation and Hygiene (KESH) Policy, which was developed in 2016 before the move of sanitation to MOWSI, specifies the MoH as the lead agency for environmental sanitation and hygiene (Ministry of Health 2016). This overlap between the ministries was evident from discussions with key informants who noted the division of responsibilities of hygiene between the two ministries.

The Ministry of Health is responsible for software, and the Ministry of Water is responsible for management of sanitation and that mainly encompasses hardware. The two [ministries] should be working closely [Policy Expert]

Development of the reviewed policies was led by the MoH (*n* = 11), by the MoH in collaboration with other ministries (*n* = 1), solely by the MOWSI (*n* = 2), or by other ministries (*n* = 2)

Other mentioned ministries included in the development of the policies include the Ministry of Education (MOE) for school hygiene, and the Ministry of Environment and Natural Resources for setting environmental standards. These ministries were part of the development of the KESH policy, the School Health Policy (SHP), and the Costed Kenya Rural Sanitation and Hygiene Roadmap. Other mentioned ministries who have a stake in hygiene-related policies include the Ministry of Devolution and Planning for mainstreaming sanitation and hygiene into national and County development plans, Ministry of Lands, Housing and Urban Development for supporting physical planning of towns and rural areas, and the treasury/ministry of Finance for strategic sanitation and hygiene investment and financing planning.

Other bodies that were involved in development of the several policies include Water Services Regulatory Board (WASREB) who are in charge of economic regulation of water and sanitation services, World Bank, the National Environmental Management Authority (NEMA), Water Services Trust Fund (WSTF), United Nations bodies such as UNICEF, Non-Governmental Organisations (NGOs), and Research and academic Institutions.

After development, County governments lead the implementation of the plans and policies while reporting to the national level. The KESH policy further identifies that the MoH would be responsible for monitoring all stakeholders and actors engaged in environmental sanitation and hygiene activities. The policy further recognizes that NGOs, Faith Based Organizations (FBOs), and other public and private agencies shall support government plans at national and County levels in activities such as hygiene education, promotion of affordable technologies, capacity building, and resource mobilization. The media is required to promote hygiene education and awareness creation, Community Based Organisations promote activities at the community level, development partners provide technical and financial support, and households should provide hygiene facilities and promote hygiene practices.

Interviews confirmed the involvement of these development partners in hygiene activities, including during planning and implementation.

Hygiene promotion has more than 25 organizations… Red Cross, World Vision, Plan International, AMREF, UNICEF, US-AID, Water Aid. [National level ministry representative]

During annual planning some partners are part of us and we make plans together …So some of their activities are deliberately put into our work plan, but we implement together…they provide the financial resources and we provide the human resource. [County level ministry representative]

They [development partners] help in sensitizing, providing [hygiene] facilities mostly for the vulnerable, educating, and training Community Health Promoters (CHPs) and our leaders. Most of the key persons in the community were trained on hand washing and sanitation, village elders, teachers, church leaders, and youth leaders. [County level implementation representative]

### Content

#### Legal frameworks

Although hand hygiene has not been specifically covered in any one legal or policy document, hygiene has generally been addressed in some legal instruments, including the Constitution, the Public Health Act, Water act, and the County governments Act. Collectively, these instruments establish rights, mandates, and service delivery obligations related to health, sanitation, water, and County-level planning. However, they do not provide detailed standards or implementation requirements specific to hand hygiene.

The Constitution of Kenya recognizes the importance of access to clean water and sanitation facilities as a basic human right. It provides that every person has the right to the highest attainable standard of health, reasonable standards of sanitation, and clean and safe water in adequate quantities. It also requires parliament to enact legislation that ensure equitable sharing of water resources among all citizens, and obligates the national government to ensure equitable sharing of resources for basic service provision, including water and hygiene facilities in all areas-including marginalized areas.

The Public Health Act provides a legal basis for promoting health and preventing hygiene-related diseases (Goverrment of Kenya 2012). While the Act addresses sanitation such as sewage disposal and waste management; references to hand hygiene are limited to specific contexts such as handling milk and dairy products. The Act provides for regulations for food handling to protect the health of the public, especially among food handlers (Section 134). The Water act does not explicitly mention hand hygiene but reinforces the legal conditions necessary for hand hygiene by affirming the right to clean and safe water in adequate quantities, and to reasonable standards of sanitation (Article 63) (Republic of Kenya 2015).

The County Government Act establishes the role of County Governments in service delivery and local development planning. County governments are required to plan for well-balanced settlements, ensure productive use of resources such as water (Article 103), develop 5-year County integrated development plans (Article 108), and ensure delivery of services (Article 116).

#### Policy frameworks

As with the legal framework, there was no single policy instrument that focused on hand hygiene; instead, hand hygiene appeared in several policy frameworks covering environmental sanitation, infection prevention, and rural sanitation; a sentiment that was confirmed by the key informants.

We may not have a specific policy on hand washing, but there may be regulations or proposals that directly or indirectly influence hand washing. [Legal expert]

- Definitions, critical moments and process of hand hygiene

Hygiene is incorporated into policy frameworks on environmental sanitation and water, and often through the mention of hygiene. Few documents (*n* = 5) defined hygiene or hand hygiene. The KESH policy, the menstrual hygiene management strategy and policy, the Costed Kenya Rural Sanitation and Hygiene Roadmap, and The National ODF Kenya 2020 Campaign Framework defines hygiene, while the KESH policy defines and categorizes food and personal hygiene. A consolidated definition of hygiene from all these documents is ‘practices such as handwashing, food hygiene, and menstrual hygiene management that maintain cleanliness, health, and healthy living and which prevent disease spread’. The KESH policy further recognizes personal hygiene as keeping the body clean to prevent disease, environmental hygiene as the maintenance of a clean and healthy environment to prevent disease, and food hygiene as keeping food clean and safe in the entire pre-consumption chain in order to prevent disease. The policy also bundles hygiene onto sanitation, and defines improved sanitation and hygiene as encompassing proper management of human excreta, personal, domestic, public, and food hygiene; solid and liquid waste management, health care waste, and protection against vectors and rodents. The National Sanitation Management Policy (NSMP) identifies hygiene as a social guiding principle and mentions handwashing and food hygiene as components of sanitation and hygiene (MoWSI 2024).

Critical hand hygiene moments are not defined across reviewed policy documents. However, documents imply key moments through activities associated with exposure to pathogens including handling of human excreta, menstrual hygiene management, and those related to food handling. The policy guidelines on control and management of diarrhoeal diseases, for example, advocates for the promotion of handwashing with soap and running water at key times, including before preparing food, before feeding the baby, after visiting the toilet, and after changing the baby's diapers (Ministry of Health 2014). Similarly, the reviewed documents generally acknowledge the importance of soap and water in handwashing, but they do not consistently specify the steps and methods of hand hygiene.

The policy frameworks do not provide standardized definition of handwashing facilities. The National ODF Campaign framework defines a handwashing facility as a fixed or movable device to contain, transport or regulate the flow of water to facilitate handwashing. Notably, the KESH policy lacks a definition of handwashing or hygiene facilities. The roadmap for rural sanitation uses the terms ‘hand hygiene facility’ and ‘handwashing facility’ synonymously, and adopts the definition of a hand hygiene facility as used by the Joint Monitoring Programme (JMP) for Water Supply, Sanitation, and Hygiene.

b. Requirements for hand hygiene

The definitions of hygiene in the various documents allude to the importance of water and soap for handwashing. As indicated, access to water is mentioned in various legal instruments that stipulate the need for access to water for all (GOK 2010, Republic of Kenya 2015).

Water, sanitation, and hygiene are critical components of Kenya vision 2030 under the social pillar and are noted as key to achieving sustainable development, health, and economic growth. Although hygiene is not mentioned, the document aims for accessibility to improved water and sanitation for all by 2030, through projects such as construction of water supply infrastructure. Stakeholders equally acknowledged and related the link between hygiene, health, and vision 2030; signifying the importance of hygiene in national development.

Health belongs to the social pillar…. how do we ensure that everybody is accessing the highest standard of sanitation because if you don't meet this, then it affects the health of the individual or the entire community. [National level ministry representative]

The Costed Kenya Rural Sanitation and Hygiene Roadmap acknowledges the basics of water and soap as defined by the JMP as being essential for hygiene, and proposes the inclusion of a handwashing facility with soap/ash and water near a latrine for an administrative unit to be declared open defecation free. The framework also puts in place mechanisms for monitoring and evaluation to track the installation and use of handwashing facilities.

The Infection Prevention and Control (IPC) strategic plan recognizes that hand hygiene facilities in health facilities should be adequate and functional, and that water should be available and in sufficient quantity. The menstrual hygiene policy recommends the inclusion of handwashing facilities for girls and women as critical elements of menstrual hygiene management.

The minimum requirements for effective handwashing are unevenly addressed across reviewed policy and legal documents. Although soap is essential for handwashing, its importance is under-represented in the majority of reviewed documents. The Costed Kenya Rural Sanitation and Hygiene Roadmap is a notable exception, as it acknowledges the importance of soap, which is specified in the definition of hygiene facilities, in plans for ensuring access to hygiene facilities, and in costing for hygiene. None of the reviewed legal or policy instruments provides explicit guidance on drainage or wastewater management specifically for hand hygiene facilities. However, 6 policy documents (the KESH policy, Public Health Act, SHP, Costed Kenya Rural Sanitation and Hygiene Roadmap, National Water and Sanitation Services Strategy, and the Rural Sanitation and Hygiene protocol) mention wastewater management, liquid waste, and grey water that may include hand hygiene waste water. Similarly, Only the IPC Strategic Plan (2021–25) mentions use of alcohol-based hand rub as an alternative to handwashing, and no policy instruments provides guidelines for hand drying as part of hand hygiene.

c. Hand hygiene in different settings
  - Schools

Hygiene in schools is addressed in 6 policy frameworks. The KESH policy acknowledges the need for improved access, operation, and maintenance of handwashing facilities in schools and recommends availability of water at all times and access to child-friendly hygiene facilities. The KESH strategy recommends that sanitation and hygiene should be included as a compulsory subject in the curricula. The SHP recognizes the importance of adequate and well-maintained handwashing facilities with soap, and that they should be placed strategically within the vicinity of the toilet/latrine, eating areas and playgrounds. The policy makes reference to approved architectural drawings of WASH facilities including handwashing facilities. The policy further tasks the County Health Departments to monitor the functionality of WASH facilities in schools, and some of the indicators include ‘percentage of schools with adequate and appropriate handwashing facilities with soap’ and ‘percentage of schools promoting hand washing at critical times’. The Costed Kenya Rural Sanitation and Hygiene Roadmap targets that all the schools in rural areas would have basic hand hygiene facilities by 2030. The menstrual hygiene policy recommends for separate toilets and handwashing facilities for girls and women, with provisions for the safe disposal of menstrual waste. Similarly, the NSMP calls for schools to have adequate and safe water supply, sanitation, and hygiene facilities.

ii. Hygiene in institutional settings

Hygiene in institutional settings is provided for in 2 frameworks. The KESH policy defines institutions as industries, factories, and garages; hotels, restaurants, and guest houses; worship centres; nursing homes, orphanage, and elderly care centres; police and military camps, prisons, refugees, or internally displaced persons camps; office premises; and higher institutions of learning. The public health Act and the KESH policy and framework recognize the importance of hygiene and recommend the provision of hygiene facilities in all these institutions, including in the initial construction plans.

iii. Health facilities

Hand hygiene in health facilities is highlighted in 4 policy frameworks. The KESH policy and the IPC strategic plan recognize the importance of hygiene in health facilities and in prevention of infections; and calls for the provision of adequate and functional facilities. The KESH policy also mentions the need for maintenance of the hygiene facilities. The IPC Strategic Plan further specifies that the facilities should have adequately displayed posters. The Costed Kenya Rural Sanitation and Hygiene Roadmap targets that all health facilities in rural areas would have basic hand hygiene facilities by 2030 and advocates for deploying of handwashing facilities at health care facility entrances. The NSMP call for health facilities to develop and implement safely managed sanitation and hygiene management plans.

iv. Public spaces

Hygiene in public spaces is specified in the KESH policy, which defines public spaces as areas or places open and accessible to all citizens, including markets, trade fairs, and recreational areas (playgrounds, beaches, recreational halls), transport facilities/stands/stop-over locations such as railway stations, passenger trains, bus stop-over locations, bus stands, airports, harbours, fishing camps and fish markets, informal mining camps, burial places, depots/warehouses, yacht clubs and beaches, solid waste disposal sites, fuel stations, and public toilets. The policy calls for the provision of adequate, reliable, and accessible handwashing facilities, as well as operation and maintenance of these facilities by the County governments. The policy also directs plantation and campsite owners to provide safe drinking water, hygienic toilet facilities, and handwashing facilities with water and soap for workers or clients working in or visiting the plantations/camps. The Community health policy, Menstrual Hygiene Policy, Costed Kenya Rural Sanitation and Hygiene Roadmap, the ODF 2020 Campaign Framework and the Rural Sanitation and Hygiene protocol mention hand hygiene in public places or spaces without further elaboration.

v. Domestic spaces

Domestic spaces are covered in three policy documents. The KESH policy combines both urban and rural areas by specifying that households in rural areas and landlords in peri-urban, slum settlements, as well as small urban and market canters have the responsibility to invest in improving their sanitation and hygiene. The Costed Kenya Rural Sanitation and Hygiene Roadmap, and the national ODF campaign Framework only provides for the provision of handwashing facilities among households in rural areas. Hand hygiene and handwashing facilities are covered under the context of implementation of the national ODF campaign Framework.

vi. Disaster and emergency situations

The KESH policy specifies that the government and stakeholders should protect the health and safety of those affected by disasters and emergencies. The national government should make available a disaster management protocol and all stakeholders involved shall coordinate to reduce the impact of the disasters. The policy specifies that the areas of focus should include—among others—handwashing with soap and hygiene promotion. Hygiene in humanitarian settings is largely missing in other documents.

d. Strategies for effective hand hygiene
  - Capacity building, education, and training

The KESH policy recognizes the role of school clubs and incorporation of hygiene education into the school health programme for continued learning among school children on hygiene, as well as training of staff in various institutions on hygiene. The NSMP calls for hygiene education in school health programmes. Capacity building is also mentioned in the IPC Strategic Plan that encourages inclusion of hand hygiene in the pre-service and in-service training curricula for all health care workers, and regular refresher courses and updates on infection prevention and control practices, including hand hygiene. The Costed Kenya Rural Sanitation and Hygiene Roadmap advocates for training staff at the health care facilities on hygiene, in addition to the provision of hygiene facilities.

The Community Health Policy and the ODF framework recognize CHPs as key agents in delivery of hygiene messages, including handwashing with soap- within the community. CHPs are tasked with health education at the household and community level, including conducting handwashing demonstrations to promote hand-hygiene practices.

The use of CHPs was confirmed from the interviews with key staff involved in implementation of hygiene interventions. The role of CHPs also encompasses collecting relevant data from the community, including data on hygiene.

We use the CHPs to educate the community and households so that they can understand the importance of handwashing. [County level implementation representative]

CHPs provide the data because it is actually what is on the ground. [County level implementation representative]

ii. Behaviour change

The Costed Kenya Rural Sanitation and Hygiene Roadmap and the National ODF framework specifies that behaviour change approaches such as social and behaviour change communication can be used to promote hand hygiene, especially in rural areas. The documents further highlight leveraging emotions such as disgust, nurture, and social status in hand hygiene campaigns and global events such as handwashing days. Key informant interviews also highlighted behaviour change approaches through global events.

On global handwashing day, we develop messages and send to all the Counties. This is raising awareness about hand washing… demonstrations are done, posters are sent out, some people have music, or digital platforms. [National level ministry representative]

Behaviour change is widely recognized in the KESH policy, the NSMP, and the IPC strategic plan. The NSMP calls for hygiene and behaviour change through information, education and communication materials. The KESH policy recommends that schools should be used as focal points for students to be agents of behaviour change.

iii. Financing

The constitution directs the national government to plan for adequate resources and financing for water, sanitation and hygiene. The KESH policy acknowledges that hygiene promotion is the responsibility of the County Governments, with the Costed Kenya Rural Sanitation and Hygiene Roadmap specifying that the funding will be under the ‘promotive health budget line’. The roadmap also specifies that County governments should enable households to purchase their own hand hygiene facilities by ensuring that facilities are available at local stores and households have access to water for handwashing. Households have the responsibility of investing in their own hand hygiene facilities from microfinance institutions, savings groups, investment clubs, and other credit schemes. To address the challenges of individual and household vulnerability due to extreme poverty, disability, or age, the policy recommends that the government should provide targeted public subsidies to affected households, but which must be kept to a minimum to avoid unsustainable demands. Stakeholders from KIIs suggested various financing mechanisms including support from non-governmental organizations and using market-based approaches (rather than subsidies) at the household level.

A market-based solution that has some sense of ownership from the community is what would provide a sustainable solution because communities still pay for other services, whether electricity or water. [Operations and implementation representative]

According to the KESH policy, financing for sanitation in schools should be provided by the Ministry of Education through schools’ budgets and parents contributions. Moreover, the national roadmap specifies that the Schools Board of Management should set aside funds for maintenance of handwashing facilities, or schools can obtain money or soap from parents. Schools are advised to include soap in their budgets and encourage parental contributions to maintain handwashing stations. A comprehensive overview of the reviewed policy documents that includes their objectives, implementation guidance and financing provision is presented in Table 2.

### Challenges

The review and interviews identified financing as a central constraint to hand hygiene implementation. The Costed Kenya Rural Sanitation and Hygiene Roadmap notes that the MoH did not have a separate budget line for sanitation and hygiene, despite estimating that achieving basic hygiene for all requires US$474.74 Million. In the absence of a dedicated budget line, hygiene activities are often bundled into broader water, sanitation or promotive health programmes. This makes it difficult to track how much is allocated to hand hygiene specifically, how funds are spent, and whether financing reaches the County and facility levels where implementation occurs. This challenge in financial allocation and spending was alluded to by key informants.

Because we lacked a sanitation department, we didn't have a sanitation code within the finance management information system. So, if you wanted to track money spent on WASH, there was a possibility that you wouldn't find it. [Policy expert]

The absence of a clear financing mechanism contributes to dependence on development partners and non-state actors for hygiene activities. While partner support is important, reliance on external financing can create uneven implementation across counties and limit the sustainability of hand hygiene interventions when partner funding is unavailable. As one County-level ministry expert noted:

Many Counties rely on partners… We do not have adequate finances. We work with most partners to promote handwashing if we don't get any [funds] from the government. [County level Ministry expert]

A second governance challenge relates to the translation of national policy provisions into County-level implementation. Key informants described a disconnect between policy documents and implementation plans, often linked to weak intergovernmental coordination, limited awareness of national policy provisions at County level, and the prioritization of other politically visible needs. This suggests that the issue is not only whether policies exist, but whether they are sufficiently embedded in County plans, budgets, monitoring systems and service delivery priorities. Key informants alluded to a disconnect between policy documents and implementation plans, often due to a lack of understanding between national and County governments, or prioritization of other aspects that were deemed more important.

Implementation is a challenge. There's inconsistency between what the documents say, versus what is done. For example, a policy might prioritize X, but the County governments prioritize Y. For example, in the KESH policy, we have provisions for how sanitation and hygiene should be provided but the implementers don't make reference to that document and the focus is usually on administrative things, or on what politicians think the citizen needs most. [Policy expert]

We have not had very good inter-governmental relations, certain things are created in the national government, and the County government have no idea what those are. [Policy expert]

A third challenge is the limited availability of routine and nationally representative hand hygiene data. Key informants noted that existing data is often fragmented, generated by specific organizations or research projects, and insufficient for establishing a comprehensive national picture. This weakens evidence-informed planning, resource allocation and accountability.

We do not have country baseline information, and when it's there, it is when organizations do research, thus we [only] have pockets of information. Some research has been done but we cannot say that [this research] gives us the country status… [National level ministry representative]

Nationally, we are using the Kenya Demographic and Health Survey (KDHS) 2016 data…which might be outdated. The 2019 census did not have handwashing. [National level ministry representative]

Despite these gaps, key informants described ongoing efforts to strengthen hygiene monitoring systems. These include expanding real-time monitoring systems, upgrading Community-Led Total Sanitation monitoring platforms to include hand hygiene indicators, and linking community-level data to the Kenya Health Information System

We are expanding the real-time monitoring system so that we can capture data with many indicators. [National level ministry representative]

The monitoring system was focused on the ODF coverage in our communities. Hand hygiene was not a main focus. We realized we have a gap…we are upgrading the current monitoring system …We will now have aspects to do with hand hygiene. [National level ministry representative]

We are working with UNICEF and National Ministry of Health to develop a platform based on the real time monitoring information system for Community Led Total Sanitation (CLTS). We have data from the community [which] we put into our ministry health information system now called Kenya health information system. [County level ministry representative]

These financing, coordination and data gaps translate into practical implementation challenges. Informants reported inadequate handwashing infrastructure, theft and vandalism of facilities, and irregular availability of soap and water. These challenges show that hand hygiene practice is shaped not only by individual knowledge or motivation, but also by the reliability of the enabling environment.

Handwashing facilities are not there and even when they are there, they are in very bad shape. You may get handwashing facilities, but there's no water, and no soap. [National level ministry representative]

People know they should wash hands, and they would wash hands if provided with the right conditions; so, it might not be lack of motivation, sometimes we have barriers of cost, time, and priorities. [Operations and implementation representative]

## Discussion

Effective implementation of hand hygiene as a preventive public health measure requires clear, enforceable policies and guidelines. The lack of such comprehensive national frameworks in Kenya creates ambiguity and coordination gaps, which potentially undermines strategic oversight and sustainability of hand hygiene interventions. The study contributes to the literature by identifying three categories of hand hygiene policy gaps in Kenya: Institutional and coordination gaps reflected in overlapping mandates between ministries and linkages between national policy processes and County implementation; definitional and content gaps reflected in the absence of a common definition of hand hygiene and inconsistent specification of hand hygiene requirements, and implementation-system gaps reflected in weak budget visibility, limited routine data, inadequate infrastructure, and insufficient monitoring indicators. These findings suggest that policy gaps are in content, governance, and systems produced by the interaction of policy context, actors, content, and process. In terms of context, the devolved system of government in Kenya separates policy formulation at the national level and implementation at the County level. Such a system aims for County-level implementation of policies with support from stakeholders in hygiene promotion and which can lead to tailored interventions that address community realities. However, without an accountability framework, such a system may have implementation challenges at the County level, especially in prioritization of activities that should be implemented. Hand hygiene is a behavioural intervention whose outcomes are often long-term, and as such County leaders may prioritize more tangible infrastructure projects especially if they lead to political goodwill.

A wide array of actors suggests a supportive environment for implementation of hand hygiene activities. These actors may fill implementation gaps but their activities may remain project-based if they are not fully integrated into County plans, budget codes and reporting systems; suggesting that such an environment works best with a coordination framework. As alluded in other studies, such a framework may encompass top-down and bottom-up approaches which should be well harmonized, and without which there may be duplication of efforts and interventions that may not be scaled up (Croese et al. 2021). This scenario highlights a lesson for hand hygiene policy implementation in Low- and Middle-Income Countries (LMICs), that development of centralized policies requires institutionalized sub-national governance, accountability and coordination frameworks that support implementation.

Financing emerged as a key mechanism through which fragmentation is replicated. The lack of a clear budget line specific to hygiene, and the even weaker visibility of hand hygiene within WASH expenditure, means that hand hygiene is vulnerable to underfunding even where it is recognized in policy. This issue is prevalent in African nations, resulting in poor capacity within governmental agencies and limited staff commitment to policy adherence (Owho and Ndakara 2022, Snyder et al. 2025). In Kenya, devolution means that County level prioritization and resource allocation may not match national goals, and partner support may further reinforce uneven implementation if it is not embedded in public finance systems and accountability mechanisms. Furthermore, progress in hygiene policy formulation and implementation across Africa is frequently hindered by corruption and a lack of political will, even when resources are available (Owho and Ndakara 2022, Ansah et al. 2024). Our findings suggest that financing affects governance and data availability within the broader health systems. Hand hygiene compliance indicators, therefore, should be integrated into routine health management information systems. Kenya and other LMICs, should establish transparent systems that link expenditure to specific hygiene-related outcomes, and which could transform hand hygiene into a funded and accountable aspect within health systems.

Results also indicate a lack of national level data, a weak integration of hygiene indicators in routine data collection systems, and inconsistent monitoring and evaluation to support decision-making. The challenge of lack of data on hygiene cuts across various countries in Sub-Saharan Africa. On the one hand, research studies give more prominence to water and sanitation (with little spotlight on hygiene), and on the other hand, there is little data on hygiene making it challenging to monitor progress and forecast progress towards achieving the set goals (Owho and Ndakara 2022, Atangana and Oberholster 2023, Okesanya et al. 2024, WHO and UNICEF 2025a). Lack of routine measurements means there is less likelihood of financial allocation, reporting or enforcement. This gap is also tied to a historical and systemic bias within the health system. Hygiene cuts across various spaces, and over the years, the focus of hand hygiene has leaned towards infection prevention and control in hospital settings and among health care workers for the prevention of hospital acquired infections (Loftus et al. 2019). As such, and as was evident in our study, there are a few guidelines and evaluations on the compliance and effectiveness of hand hygiene in health care settings with little on other settings such as domestic or humanitarian settings (Choe et al. 2020, MacLeod et al. 2023). This disparity leads to a critical lesson that Kenya contributes to the global health policy conversation: leaving out other settings such as low-income settlements and humanitarian settings without specific guidelines may reinforce inequalities, and slow down progress towards the achievement of set goals such as the SDGs. This challenge requires that hand hygiene guidelines should be supported by cross-sectoral integration of hygiene indicators into routine data collection to enable accountability.

In terms of content, results indicate a lack of policies or guidelines specific to hand hygiene, and lack of a common definition of hygiene and hand hygiene. This is not peculiar to Kenya as it has been highlighted as a common problem in Africa (Owho and Ndakara 2022). However, Kenya's case shows how such definitional ambiguity has practical governance consequences. When hygiene is bundled into sanitation or water policy without precise standards, it becomes difficult to assign accountability, cost activities, procure appropriate facilities, monitor performance, or protect hand hygiene from not being prioritized within broader WASH programming. This finding strengthens the case for national hand hygiene definitions and standards that are linked to specific settings and implementation responsibilities. As noted in other studies, it is crucial to implement clear regulatory frameworks and coordination mechanisms, as well as system-strengthening approaches that could foster coherence of policies, decision-making, identification of long-term policy impacts, and sustainable and resilient national systems (Auriacombe and Van der Walt 2021, Esteves Mills et al. 2023, Anghileri et al. 2024, Okesanya et al. 2024).

Interventions for improving overall health caused by inadequate hand hygiene focused on various approaches including behaviour change and capacity building. Such approaches, especially in Africa, have increased awareness on hand hygiene, but adopting comprehensive policies is crucial in improving overall health and well-being (Irehovbude and Okoye 2020, Akinsulie et al. 2024). Hand hygiene therefore should be addressed holistically in a health systems strengthening approach that accounts for the complex interdependence of governance, finance, infrastructure, and socio-behavioural dynamics. Additionally, water and sanitation are ‘tangible’ goods and services which are easy to include in development plans and guidelines; hygiene, however, is a personal behaviour that depends on the availability of necessary infrastructure such as water supply and sanitation services and is influenced by factors such as knowledge, individual and social norms, and beliefs (Budge et al. 2022, Ezezika et al. 2023). Sustainable hand hygiene therefore requires provision of basics such as regular water supplies and sanitation services especially in under-reached areas. A systems approach is therefore needed, where behaviour change and capacity building are linked to infrastructure, financing, institutional accountability and monitoring (WHO and UNICEF 2025b).

In terms of limitations, this study relies on primary data from key informants and secondary data from document analysis to evaluate the hand hygiene policy landscape in Kenya. The study did not measure behaviour change or health outcomes. The key informant sample was relatively small and was identified through stakeholder networks. The sample was designed to capture diverse roles across national, County, regulatory, and implementation actors, but it may not represent all perspectives within Kenya's hand hygiene landscape. The findings, therefore, should be interpreted as an analysis of the national policy and governance environment rather than a complete assessment of implementation practices within the Country.

## Conclusion

This analysis from Kenya offers critical lessons to broader health policy, decentralized governance, and system-strengthening theories. Our study demonstrates that political devolution has to be accompanied by institutionalized alignment, and that health systems should be strengthened by ensuring standardized definitions and guidelines, integration of all contexts, appropriate implementation at sub-national levels, and establishment of accountable data-driven budgeting frameworks that treat hand hygiene as a critical pillar of public health. These lessons call for specific recommendations at different levels. As a high priority, the Ministry of Health should consider gazetting national hand hygiene standards with clear technical definitions and specifications across settings. These are foundational, within the mandate of the MoH, have a lower financial implication, can be feasibly implemented, and would serve as the foundational benchmark and quality control measure required before enforcement and tracking can be implemented. After the foundational priorities, the MoH at the national and County levels should Integrate hand hygiene indicators into health information and tracking systems and develop County scorecards. Such would leverage existing digital health platforms and may require additional capacity building for staff to ensure accurate data capture. Such platforms would generate the empirical evidence needed to hold County governments accountable and justify budget requests. In the long term, the MoH, the MoWSI, in conjunction with the Ministry of finance and the Council of Governors should aim to establish a dedicated hand hygiene budget code at national and County government levels. Such requires planning with the relevant stakeholders and if feasible, would lead to dedicated resources for hand hygiene interventions.

## Data Availability

The data underlying this article are available in the article and in its online supplementary material.

## Reflexivity statement

The study has a multidisciplinary team of authors who had a contextual understanding of the Water, Sanitation, and Hygiene (WASH) environment in Kenya. The team comprised expertise in public health, sociology, and governance development, which enabled understanding and cross learning among the researchers. This combination of expertise enabled identification of documents and thorough analysis of the policy environment. The researchers’ expertise and institutional affiliation also facilitated stakeholder identification and acceptance for interviews. Even with the researchers’ background, the study was a learning process for the study team, enhancing their awareness and understanding of structural and implementation gaps of the hygiene policy environment in Kenya. To minimize bias, a collaborative approach was employed, as the researchers participated in data analysis and held several discussions to collectively review and discuss the analysis plan, which ensured that multiple lens were applied in the analysis.
